# Strategies for developing and optimizing cancer vaccines

**DOI:** 10.12688/f1000research.18693.1

**Published:** 2019-05-13

**Authors:** Hoyoung M. Maeng, Jay A. Berzofsky

**Affiliations:** 1 Vaccine Branch, Center for Cancer Research, National Cancer Institute, National Institutes of Health, Bethesda, MD, 20892, USA

**Keywords:** cancer vaccine, tumor antigen, immunotherapy

## Abstract

With the spotlight on cancer immunotherapy and the expanding use of immune checkpoint inhibitors, strategies to improve the response rate and duration of current cancer immunotherapeutics are highly sought. In that sense, investigators around the globe have been putting spurs on the development of effective cancer vaccines in humans after decades of efforts that led to limited clinical success. In more than three decades of research in pursuit of targeted and personalized immunotherapy, several platforms have been incorporated into the list of cancer vaccines from live viral or bacterial agents harboring antigens to synthetic peptides with the hope of stronger and durable immune responses that will tackle cancers better. Unlike adoptive cell therapy, cancer vaccines can take advantage of using a patient’s entire immune system that can include more than engineered receptors or ligands in developing antigen-specific responses. Advances in molecular technology also secured the use of genetically modified genes or proteins of interest to enhance the chance of stronger immune responses. The formulation of vaccines to increase chances of immune recognition such as nanoparticles for peptide delivery is another area of great interest. Studies indicate that cancer vaccines alone may elicit tumor-specific cellular or humoral responses in immunologic assays and even regression or shrinkage of the cancer in select trials, but novel strategies, especially in combination with other cancer therapies, are under study and are likely to be critical to achieve and optimize reliable objective responses and survival benefit. In this review, cancer vaccine platforms with different approaches to deliver tumor antigens and boost immunity are discussed with the intention of summarizing what we know and what we need to improve in the clinical trial setting.

## Introduction

Cancer vaccines have been extensively researched in both animal models and humans over the past 30 years across many different types of cancer. With continued attempts to develop effective cancer vaccines, several agents are licensed or were granted Orphan Drug Designation. However, the limitation in demonstrating clinical response has led to US Food and Drug Administration (FDA) approval of only one agent, sipuleucel-T, a cancer vaccine that is used to treat metastatic castration-resistant prostatic cancer in a limited group of nearly asymptomatic patients, indicating an apparent unmet need ^1^.

Cancer vaccines are highlighted in the era of cancer immunotherapy as they can induce immune responses in “cold” tumors that do not appear immunogenic on their own, potentially converting them to “hot” tumors amenable to checkpoint blockade therapy. Hot tumors are defined as ones in which the tumor itself has induced an immune response of infiltrating T cells that are not able to function because of various checkpoints such as PD-1, CTLA-4, LAG3, TIM3, TIGIT, or other immunoregulatory mechanisms involving regulatory cells (regulatory T cells, myeloid-derived suppressor cells (MDSCs), M2 macrophages, regulatory natural killer (NK) T cells, and so on) or regulatory cytokines (transforming growth factor-beta [TGFβ]), interleukin-10 (IL-10), and IL-13) ^2, 3^. A cold tumor is one that is not sufficiently immunogenic to induce such infiltrating T cells on its own, or has excluded these cells. Without a T-cell response, checkpoint blockade has no immune response to augment. Cancer vaccines can fill this gap by inducing T-cell immunity that then can be made more effective by overcoming the inhibitory signals through checkpoint blockade ^4– 9^ or other methods to overcome other immunosuppressive mechanisms. For example, blockade of TGFβ has been found to complement the effect of PD-1 blockade in enhancing cancer vaccine efficacy ^10^, perhaps because it allows entry of T cells into the tumor ^11, 12^. Anti-TGFβ proved safe in a phase I clinical trial and even showed some evidence of clinical activity in patients with melanoma ^13^, so it could be combined with anti-PD-1. Thus, understanding the difference between the cancer vaccine platforms is crucial in maximizing the synergistic effect with other immunotherapeutics and anti-cancer therapies.

## Shared tumor antigens versus neoantigens

For most of their history, cancer vaccines have focused on “shared” tumor antigens, meaning antigens that are common to most cancers of a given histological type ^14, 15^. A decade ago, a working group was assembled to develop a list of the highest-priority shared tumor antigens ^16^. These include antigens like hypoglycosylated MUC1, Wnt1, HER2, MART1, gp100, and tyrosinase. Many of these have shown promising early results but none has been licensed yet as a vaccine. For example, hypoglycosylated MUC1 was found to be a tumor antigen common to many adenocarcinomas ^17^, and clinical trials have shown promise ^18^. HER2 is known to be a surface antigen that is also a driver oncogene product constitutively signaling the cell to divide. Monoclonal antibody therapy against HER2 is a mainstay of treatment for HER2 ^+^ breast cancer and some other HER2-expressing cancers, but no vaccine that induces such antibodies is clinically available. However, peptide vaccines targeting HER2 have been successful in inducing immunity in patients with breast and ovarian cancer ^19, 20^. An ongoing trial of a dendritic cell (DC) vaccine transduced with an adenovirus expressing the extracellular and transmembrane domains of HER2, which cures mice of HER2 ^+^ tumors by inducing antibodies, is resulting in some complete and partial responses and stable disease in close to 50% of vaccinated patients with advanced metastatic HER2-expressing cancers who have failed standard therapies (Maeng *et al*., manuscript in preparation). Another shared antigen, TARP ^21^, expressed in about 95% of prostate cancers of all Gleason types and about 50% of breast cancers, has shown promise as a peptide vaccine in HLA-A*0201 ^+^ patients with stage D0 prostate cancer by reducing the tumor growth rate as measured by prostate-specific antigen (PSA) slope in about 74% of vaccinated patients at 1 year ^22^.

In the past 5 years or so, neoantigens have been found to represent a substantial portion of tumor antigens. These are antigens that are created by point mutations, insertions, deletions, or translocations that produce novel amino acid sequences present only in the tumor and not normal cells. Some fraction of these can be presented by HLA molecules of the patient and thus constitute neoepitopes of neoantigens. It was found that the response rate to checkpoint inhibitors correlated with the number of such mutations (tumor mutational burden) or predicted neoepitopes ^4^. For this reason, multiple groups are developing strategies to identify such neoepitopes in each patient’s cancer and then synthesize a personalized vaccine to treat his or her cancer ^23^. One of the first attempts at making a neoepitope vaccine, before the term was coined, was the targeting of mutant epitopes created by point mutations in Kras or p53 that commonly occur in cancers. In a vaccine trial of such personalized mutant ras and p53 peptides, the cancer patients who made an interferon-gamma (IFNγ) T-cell response to their own mutant peptide vaccine had a median overall survival more than a year longer than patients who did not make such a T-cell response ^24^. Now that the technology to rapidly identify such mutations has matured, more such approaches are under way. It should also be noted that not all neoantigens are of equal quality for the induction of anti-tumor immunity; variation in quality depends on factors such as affinity for the patient’s HLA molecules, frequency of relevant T-cell receptors (TCRs) in the repertoire (which may be affected by cross-reactivity with microbial antigens), and expression levels in the tumor ^25– 28^.

## Vaccine platform types

Developing preclinical models for cancer vaccines has been difficult as immune systems of animals and humans are different; more importantly, mouse tumor models do not often mimic the human disease that presents clinically when already established. Despite the challenges, each translation of cancer vaccines to the clinical setting has yielded a deeper understanding of each platform. In this article, we will focus on those with successful translation to clinical trials, reviewing the historical changes to develop vaccines that are more effective.

**Figure 1.**
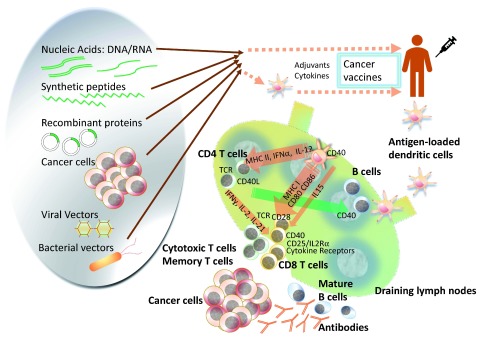
Cancer vaccine platforms and interactions in the immune system. Antigen-specific cancer vaccines come in a variety of compositions to deliver the tumor antigen of interest. Compared with cell-based vaccines, the rest of the platforms have the merit of potential for simpler manufacturing and flexibility in delivery of the vaccines along with special features of individual platforms. Cell-based vaccines, as represented by dendritic cell (DC) vaccines, have an advantage in that investigators can perform targeted loading of the antigen of interest, circumvent poor DC maturation in patients with cancer, and manipulate *in vivo*, but standardizing the manufacturing and quality assessment is another hurdle to surmount. As the network of the immune system is unveiled further, more efficient and intelligent design of cancer vaccine platforms will expand the territory to use these either by themselves or in combination with other cancer therapy options in this era in which cancer immunotherapy has become the fourth pillar in oncology, beyond surgery, chemotherapy, and radiation. IFN, interferon; IL, interleukin; MHC, major histocompatibility complex; TCR, T-cell receptor.

Cancer vaccines can be categorized in a few different ways. One way to classify cancer vaccines is based on the biologic formulation or antigen source of the vaccine: nucleic acids, peptides, recombinant proteins, microbial vectors, whole tumor cells either autologous or allogeneic, manipulated antigen-presenting cells (APCs), and other artificial systems ( Figure 1). The other major way is based on the type of cancer antigens, the methods of antigen selection, or uniqueness of the antigen (for example, shared versus neoepitopes). There have been controversies about whether an oncolytic virus should be considered a cancer vaccine or not. Strictly speaking, oncolytic viruses that are intended to induce direct cytopathic effects as the main mechanism of action are different from cancer vaccines, even though some of the post-treatment immune reactions might initiate similar immunologic cascades, turning the tumor cells into “endogenous vaccines” ^29^. The route and mode of delivery are also important topics but should be addressed in the context of an individual vaccine formulation to understand why certain routes may or may not work better for a particular vaccine or formulation.

### 1. Nucleic acid/DNA/mRNA vaccines

The use of nucleic acids with the hope of developing gene therapies to correct any mutations or deficiencies or vaccines to induce specific antibody or T-cell responses against a pathogen (prophylactic vaccines) or a tumor antigen (cancer vaccines) was one of the highlighted fields in biomedical advances in 1990s. The concept of using non-live vaccines offers an opportunity to induce both humoral and cellular immunity without any concern of introducing infectious organisms that can potentially cause the illness in the vaccine recipients or unintended individuals.

Typically, a DNA vaccine is prepared by inserting the gene sequence of interest into a plasmid backbone that needs to be expanded and purified for administration via intradermal, subcutaneous, or intramuscular routes ^30^. When the plasmid enters the residential APCs or surrounding cells (such as myocytes), transcription occurs, resulting in expression of the protein of interest. The cell machinery provides post-translational modifications to the antigen in a manner similar to that in target cells of the vaccine. APCs play a dominant role in the effect of DNA vaccines by presenting the processed peptides on major histocompatibility complex (MHC) class I molecules either from a direct transfection or through cross-presentation of antigens taken up by phagocytosis of transfected cells ^31^. Also, secreted tumor antigens are processed by the endocytic pathway. Then antigen-loaded APCs travel to a draining lymph node (DLN) to present the antigen to naïve T cells with co-stimulatory molecules to induce T-cell expansion, cytokine secretion, or interaction with B cells. If the vaccine is administered intramuscularly, transfected muscle cells can also present the peptide-MHC complexes, increasing the chances of T-cell expansion, although preclinical evidence suggests that the relevant presentation is primarily by professional APCs in the DLNs ^32^. Another approach is to use DNA priming followed by a boost such as modified vaccinia Ankara (MVA) or a canarypox vector, as was researched originally in HIV vaccines ^33, 34^. Variations in delivery methods, including electroporation, that can deliver the plasmid along with other elements (such as toxoids or cytokines) and the use of xenogeneic DNA to enhance immune response have been studied and translated to clinical trials in solid tumors, including melanoma and prostate cancer ^35– 39^. In veterinary medicine, DNA vaccines targeting cancer have been licensed in a number of solid tumors. Accumulated information from the animals may contribute to the development of next-generation vaccine platforms ^40, 41^.

Even though DNA vaccines have shown immune responses in humans, the potency is still limited. The development of novel technologies in the delivery of nucleic acids, such as hydrophobic peptide mix, gene gun, or electroporation, can be incorporated to enhance the efficiency ^39, 42, 43^. Large batch manufacturing and a long shelf life make a DNA vaccine attractive to the community practice setting, not limiting the treatment opportunities to cutting-edge research centers, in contrast to some other personalized vaccine strategies. The other significant benefit of a DNA vaccine is the versatile construction that enables the developer to include encoding of optional safety features instead of using any intact viral particles. However, integration to cellular DNA, the theoretical risk of antibiotic resistance due to selection markers in the plasmid, and the development of autoimmune conditions due to autoantibody production have yet to be addressed. Guidelines from the regulatory groups in the European Medicines Agency, the World Health Organization, or the FDA are available to address such safety concerns about developing DNA vaccines but those guidelines are focused on preventive vaccines for infectious organisms ^44– 46^.

In a study of patients with metastatic melanoma (ClinicalTrials.gov identifier: NCT02035956), Sahin *et al*. analyzed the mutated neoepitopes, prioritized by predicted MHC affinity, and treated the cancers with up to 10 neoepitope-encoding mRNAs ^47^. About 60% of the patients developed *de novo* immune responses and the majority were CD4 responses. Tumor tissue sections showed neoantigen-specific killing by vaccine-induced T cells. The authors also described objective tumor responses, decreased cumulative metastasis, and sustained survival in selected patients. This study supports a newer concept of cancer vaccines using nucleic acids.

### 2. Peptides and proteins

Synthetic peptides have been used successfully in the induction of tumor-specific T-cell responses in clinical settings against both microbials and cancer ^48^. The use of peptides compared with whole cell lysates or protein has an advantage that only the epitopes of interest can be delivered to the immune system instead of overloading immunologic pathways with irrelevant antigens that might compete with the relevant epitopes or might induce autoimmune responses. Also, using only T-cell epitopes can avoid the danger of anaphylaxis associated with antibody responses by avoiding B-cell epitopes. Sometimes, the peptides require modifications to allow enhanced binding to MHC molecules to increase the immunogenicity, a process called epitope enhancement ^49^. Often, a few amino acids are added or mutated to enhance the immunogenicity and modulate the amphiphilicity and these changes have been shown to either enhance or inhibit anti-tumor T-cell responses ^50^. Vaccines using xenogeneic peptide/protein by themselves or loaded to DCs can be a further extension of enhanced immunogenicity originating from the difference in xenogeneic amino acid sequences while having the majority in common ^40, 51^. Peptides can be encapsulated in particles such as liposomes or conjugated to adjuvants to optimize the antigen uptake by specialized APCs for the MHC class I and II presentation to CD8 ^+^ cytotoxic or CD4 ^+^ helper T cells, respectively, and such technologies have been translated to clinical trials in both hematologic (ClinicalTrials.gov identifier: NCT03349450) and solid cancers ^52– 56^. It is relatively easy to add or substitute peptides for other epitopes compared with adoptive cell therapies such as chimeric antigen receptor (CAR) T cells or TCR-transgenic T cells, which are harder to adapt to antigen loss or alteration ^57– 61^. Using synthetic long peptides (SLPs) of 15 to 30 amino acids has been advocated by some groups who propose that professional APC-processed SLP can more efficiently induce anti-tumor responses than direct presentation of shorter peptides to the cytotoxic T cells, that can be mediated by non-professional APCs that lack costimulation ^62, 63^. Melief *et al*. have demonstrated such a concept in several clinical trials focused on human papilloma virus (HPV)-associated cancers, and vaccination using SLP has been widely applied in various solid tumors ^64– 70^. At the same time, shorter peptide vaccines have also been tested clinically in HPV-related disease and have been shown to be immunogenic ^71^. Idiotype vaccination in B-cell malignancy is another unique concept of targeting tumor-specific antigens in the form of idiotype produced uniquely by the malignant B cells or plasma cells ^72, 73^. The challenge is the limited knowledge in intracellular processing and methods of tracking it in humans when the limited data are based largely on animal, especially murine, models. MHC class I processing involves endoplasmic reticulum aminopeptidases, cytosolic proteasomes, and peptidases, whereas MHC class II peptide production and processing take place in endolysosomal compartments involving cathepsin-like proteases ^74^. The process of cross-presentation allows exogenous antigens, which normally go through the MHC class II pathway, to be processed via the MHC class I pathway to trigger a cytotoxic CD8 T-cell response ^75^. Sometimes, endosomal proteases can affect epitope expression during cross-presentation ^76, 77^.

Clinical trials using shared tumor antigens have been safe and immunogenic in many studies but so far have not been successful in showing clinic benefit sufficient for FDA approval. GV1001 against a telomerase peptide (TeloVac) for advanced pancreatic cancer combined with chemotherapy failed to show improved overall survival in a phase III trial ^78^. Several other phase III trials, including studies targeting a MUC1 antigen (tecemotide, START trial) and EGFRVIIII in glioblastoma multiforme (rindopepimut, CDX-110, ACT III study), also failed to show improvement in overall survival despite high expectations when they went into advanced clinical studies ^79, 80^. The strategy to include multiple epitopes in one vaccine did not result in any superior results in advanced pancreatic cancer or renal cell carcinoma, indicating the serious need for better strategies to overcome several immunologic hurdles to control the cancer. Personalized peptide vaccines targeting neoantigens are among newer efforts to liberate trammeled immune responses against cancer ^25^.

### 3. Microbial vectors and non-bacterial human pathogens, including viruses and helminths

Microbes can function even in necrotic tissues, and the immune modulatory effect by the microbes can be an additional benefit added to the direct killing of cancer cells. Using bacteria or their products in cancer treatment goes back to 1890s, when Coley described the accidental finding of an inoperable case of sarcoma that improved with erysipelas; that led to treatment with inoculation of Streptococcus and *Bacillus prodigiosus*, now known as *Serratia marcescens* ^81^. In his reports, he described the difficulties in inducing meaningful infection from the inoculation, the clinical course after inoculation of “Coley’s toxin”, and the regression of inoperable tumor that lasted several years in one of the patients. Since then, cancer immunotherapies have used various bacteria or their products, such as *Clostridium novyi*, *Listeria monocytogenes* (Lm), and *Salmonella typhimurium* ^82– 86^. Several organisms have been tested either to serve as a vector purely to express tumor antigens or in combination with DCs or other adjuvants. In addition to those listed above, shigella and bacillus Calmette–Guérin (BCG) among bacteria, and vaccinia, lentivirus, adenovirus, yellow fever, and HPV among viruses are well-known examples of microbial vaccine vectors.

On another front, microbiome research in cancer and immunology has gained momentum in the past decade, leading to versatile approaches in using the microbiome, including the consideration of normal flora such as *Lactobacilli* as vectors for gynecologic cancer. Besides direct anti-cancer effects of the infection, infection of the tumor tissue with facultative anaerobes can enhance the antigenicity of tumors that otherwise could have been tolerogenic or poorly antigenic while driving the alteration in the immune subsets in a pro-inflammatory microenvironment ^87, 88^.

The changes in immune landscape in the use of bacterial cancer vaccines are noteworthy. MDSCs, tumor-associated macrophages, tumor-entrained neutrophils, and tolerogenic DCs are all myeloid cells that are immune-suppressive and found during tumor growth in both primary and metastatic lesions. Monocytes that are attracted to the tumor generally differentiate into macrophages, typically M2 macrophages that are characterized by arginase 1 (Arg1) expression and IL-10 known as immunosuppressive markers that contribute to immune evasion of cancer, whereas M1 macrophages contributing to anti-tumor activity express nitric oxide synthase (NOS2) and secret tumor necrosis factor-alpha (TNFα). Injection of attenuated *S. typhimurium* into HER2/neu-expressing CT26 tumors in a mouse model showed the shift to mature phenotype in macrophages in the tumor or spleen. Injection of attenuated Lm to an ID8-Defb29/Vegf-A ovarian carcinoma mouse model led to increased macrophage infiltrates in the tumor, favoring an M1 profile. These findings suggest that bacterial vaccines can reprogram the pro-inflammatory nature of cells and influence M1/M2 phenotype-specific differentiation ^89^. Melanoma cells infected with wild-type Lm can differentiate into professional APCs that express mature DC markers such as CD11c, CD40, CD83, and MHC class II ^90^. These findings suggest how pathogens can stimulate the immune function via co-stimulatory molecules involving Toll-like receptor (TLR) signaling pathways to activate killer T-cell and NK cell activities to kill tumor cells. On the other hand, tumor-associated neutrophils (TANs) are also of importance in the research of cancer vaccines using bacteria. Vendrell *et al*. reported increased recruitment of TANs after the injection of *Salmonella typhi* in a mouse model of breast cancer ^91^. TANs can alter the tumor microenvironment (TME) by secreting inflammatory cytokines such as IFNγ and TNFα that potentiate the local immune infiltrates and systemic response. Salmonella strains that are engineered to express such inflammatory cytokines have shown enhanced anti-tumor activity *in vivo*. In regard to T-cell responses, injection of attenuated Salmonella species resulted in increased Th1 phenotype CD4 T cells in tumor infiltrates, slower tumor growth, and improved host survival in mice ^92^. These effects were decreased at least in part in a T cell–depleted mouse model, suggesting the requirement for (presumably cytotoxic) T cells in the mechanism of action. Similar observations were reported with Lm and the parasite *Toxoplasma gondii* ^93^. Bacterial vaccines can further modulate the TME by decreasing the expression of PD-1 in tumor-infiltrating lymphocytes (TILs), inducing central and effector memory T cells, and reducing MDSC immunosuppressive potential. Several strains of attenuated or avirulent Lm strains such as replication-deficient Lm∆dal∆dat (Lmdd) have been tried to activate MHC class I and II pathways and also to induce antigen-specific T lymphocytes ^94^. Virulence factor listeriolysin O (LLO) protein is known to boost the immune reaction by inducing IL-12, IL-18, and IFNγ and functions as a naturally occurring adjuvant when delivering the tumor antigens ^95^ and can improve ratios of CD8 ^+^ T cells to regulatory T cells ^96^. So far, Lm-based vaccines have been brought into clinical trials for several solid and hematologic cancers, including HPV-associated cervical cancer, melanoma, breast cancer, pancreatic cancer, and lymphoma.

Aside from bacterial or yeast platforms, the viral platform of cancer vaccines has its own vast ground. Many attenuated or less virulent related viruses targeting as measles, mumps, rubella, smallpox, and polio have been used successfully to prevent or decrease the severity of viral illness by inducing protective immunity in the history of modern medicine. With the advancement of genetic engineering, these viruses can also be modified as vectors to deliver and express the antigens that will elicit immune responses even in tumors that are not immunogenic by themselves during the natural history of the specific cancer type. Viral particles can express *in situ* molecules that will serve as pathogen-associated molecular patterns to increase the possibility that a tumor-associated antigen (TAA) will be presented to the immune system. Activation of TLRs is believed to play a critical role in increased anti-tumor activity in therapies using viruses ^97, 98^. Viral vectors used in cancer vaccines can deliver the TAA, with or without co-stimulatory molecules or cytokines, that will be processed in the MHC class I pathway and produce epitopes expressed on the surface of the infected cells with MHC I, and/or with MHC class II if an APC is infected. In the case of non-lysing virus, delivery of a TAA alone might not generate enough signals for tumor cell killing. With non-lysing virus, co-delivery of a co-stimulatory molecule or cytokines can increase the chance of recruitment of professional APCs. In either case, replicating viruses at the injection site cause pro-inflammatory responses mimicking the immune response against infecting virus in non-cancerous tissues that can result in cancer cell death.

Adenoviruses, especially replication-incompetent Ad2 and Ad5, have been popular to deliver TAAs with or without co-stimulatory molecules ^99^. However, the majority of adults have developed immunity from long-term environmental exposure to adenoviruses, and the immune response and clinical effect were minimal because of pre-existing immunity at least in part. Poxviruses have the advantage as pre-existing immunity against poxvirus exists only in patients who were exposed to vaccinia virus. Viral tropism of the members of this family to directly infect APCs is another advantage ^100^. MVA is a proprietary agent that was produced by more than 500 culture passages of vaccinia in chicken embryo fibroblasts. During the passages, MVA became highly attenuated as it lost many poxviral genes and replicative potential in most mammalian cells. A recombinant MVA-expressing human 5T4 (MVA-h5T4), called TroVax for metastatic castration-resistant prostate cancer, and a recombinant attenuated vaccinia virus (rVV) vaccine or fowlpox virus (rFV) against human carcinoembryonic antigen (c-VV-CEA) with or without a triad of co-stimulatory adhesion molecules (TRICOM) are great examples that were tested in the clinical setting ^101, 102^. The TRICOM vector expresses three co-stimulatory molecules—CD54 (ICAM-1), CD58 (LFA3), and CD80 (B7.1)—co-expressed in the recombinant virus, and the triple combination was found to be more effective than any single one of these molecules ^103^. Examples of intelligently designed heterologous prime-boost strategy can again be found in a vaccinia prime–fowlpox boost, PANVAC encoding CEA and MUC1 alongside TRICOM ^104^. Because the vaccinia vector induces immunity against itself but not against the fowlpox vector, which also does not induce immunity to itself, it is possible to achieve a better response by priming first with the vaccinia vector and then boosting repeatedly with the fowlpox vector. Thus, a heterologous prime boost can be more effective than a homologous prime boost ^105^. PROSTVAC used priming with rVV-PSA-TRICOM followed by rFV-PSA-TRICOM to deter any neutralizing antibody responses and facilitate antigen-specific T-cell responses ^106^. Despite promising phase II results, a phase III randomized trial of PROSTVAC as a single agent did not meet its endpoints to show improved overall survival in planned interim analysis in patients with minimally symptomatic metastatic castration-resistant prostate cancer, and combination trials are ongoing ^107, 108^.

### 4. Dendritic cell–based vaccines

DC-based vaccines have several advantages despite their inherent logistic problems ^109, 110^. DC maturation is often defective in patients with cancer and this is due in part to cytokines such as vascular endothelial growth factor (VEGF), which induces STAT3 expression and inhibits maturation ^111– 113^. Thus, antigen presentation can be defective in these patients, resulting in poor induction of T-cell responses. DC vaccines can overcome this problem in patients with cancer by starting from DC precursors, such as elutriated monocytes, which then are matured *in vitro*, away from the negative influences of the cancer environment ^22, 114^. A second related advantage is that the DCs can be matured *in vitro* to induce higher levels of co-stimulatory molecules or MHC molecules or both, making them more immunogenic ^109, 115^. A third advantage is that viral vectors can be used to transduce the DCs to express tumor antigens without neutralization by pre-existing anti-viral antibodies ^116^.

Even though DC-based vaccination has been a popular platform to elicit immune responses, there is no consensus on how to optimize the preparation for consistent or durable responses. Also, handling individual patient’s cells can be an obstacle in reducing the cost and simplifying the delivery process. To tackle this issue, understanding the biology of DCs is essential. DCs are a heterogeneous group of specialized APCs from the same bone marrow progenitors as monocytes, called monocyte and DC progenitors, that differentiate into three major subsets of DCs: CD1c DCs, which are predominant; CD141 ^+^ DCs; and plasmacytoid DCs (pDCs) ^117– 119^. The first two are called conventional DCs (cDCs). pDCs play a major role in viral infections by rapid cross-presentation of antigens and production of up to 1000-fold more type I IFNs (α and β). Human DCs lack lineage markers but express MHC class II. Whereas cDCs can be found in almost all peripheral tissues, including lymphoid organs, pDCs are discovered mostly in T-cell areas of lymphoid organs, such as lymph nodes, spleen, bone marrow, Peyer’s patches, tonsils, thymus, and liver. DCs can activate or negate anti-tumor T-cell responses by affecting killer or regulatory T–cell activation ^120^. Mobilized CD34 precursors can be differentiated into monocyte-derived DCs (MDDCs) that yield several different types of myeloid cells, including all three major types of DCs, as a consequence of working with higher levels of progenitor cells. It may be important to manipulate each subset individually to improve immunogenicity ^121^, and some groups use bead-selected DCs. For example, in some studies, Langerhans-like DCs induced with IL-15 were found to be more effective at eliciting CD8 ^+^ T-cell responses ^122^. Moreover, activation of Langerhans cells with a cytokine combination could induce immunity against HPV that then could be boosted with a therapeutic vaccine ^123^. Similarly, the BATF3-expressing DC subset may be critical for cross-priming and for reactivating a memory anti-tumor response ^124^. On the other hand, immature DCs have been found to induce regulatory T cells ^125^.

The first and only FDA-approved cell-based therapy for metastatic prostate cancer, sipuleucel-T, contains a variety of cells, including B cells, monocytes, DCs, and NK cells, that are cultured and processed in the presence of prostatic acid phosphatase (PAP) fused with granulocyte-macrophage colony-stimulating factor (GM-CSF) for 2 days following leukapheresis in designated apheresis centers ^1, 126^. Patients receive up to three doses of freshly prepared products at 2-week intervals. The most commonly used method is to differentiate the monocytes from peripheral blood mononuclear cells (PBMCs) into DCs, so-called MDDCs. PBMCs that are monocyte-elutriated with or without CD14 bead selection are differentiated into immature CD14 ^−^CD83 ^−^ DCs by culturing in IL-4– and GM-CSF–supplemented culture media usually for 3 to 5 days followed by antigen loading for 24 to 48 hours. DCs then can be matured with lipopolysaccharide (LPS) and IFNγ or with a cytokine cocktail ^22, 109^. Matured DCs are harvested for the delivery. However, owing to the labor-intensive and costly manufacturing process, aliquoting and freezing individual doses and use of automated closed culture systems are being actively investigated. For allogeneic sources of DCs, cDCs of umbilical cord blood origin can be one option to meet the need for unrelated healthy donors. However, the immune functions of the DCs in umbilical cord are questioned and the use of HLA-half matched allogeneic DCs has not been successfully translated to the clinic yet ^127, 128^.

Several ways have been adopted for antigen loading. As DCs are professional APCs, loading with synthetic peptides, protein, tumor cell lysates, and RNAs and even direct infection with microbes are viable options. APCs fused with tumor cells, whether from an autologous or allogeneic source, were the prototype of cancer vaccine from the 1990s, when the approach was first described ^129, 130^. Despite high production costs due to patient-specific therapies, there have been continued efforts to translate DC vaccines to clinical trials in both solid and hematologic cancers ^114, 131– 133^. Using whole lysates of either autologous or allogeneic tumor cells can lead to the presentation of multiple epitopes for a prolonged period, enabling longer antigen presentation that will allow both CD4 and CD8 T-cell responses. Wild-type or attenuated pathogens can serve as vectors to carry the genes encoding TAA to be expressed in DCs. Transfection of the nucleic acids encoding TAAs can induce potent TAA-specific T-cell responses at least *in vivo*. For example, mRNA can be transfected by electroporation (renal cell carcinoma, ClinicalTrials.gov identifier: NCT01582672; advanced melanoma, phase II, ClinicalTrials.gov identifier: NCT01302496) or a cationic lipid such as DOTAP ^134– 136^. Alternatively, retroviral transduction has been used to facilitate and prolong stable TAA expression. Viral vectors such as lentivirus or adenovirus have been employed in several preclinical and clinical studies with DC vaccines because of the advantage of transducing non-dividing cells ^137– 139^. In regard to the issue of adequate involvement of CD4 cells to enhance CD8 cell response in DC-based vaccines, adding other antigens such as keyhole limpet hemocyanin (KLH) as a target for helper T cells that can facilitate the antigen presentation and migration to the DLNs is an option ^22, 115^.

As the manufacturing and quality control of the DC vaccines are highly variable, development of large-scale studies is limited and the interpretation of smaller-scale studies is often difficult ^140– 142^. Also, there is no consensus in measuring the correlatives to evaluate the vaccines. How and what cytokines or markers should be measured and in which samples? For example, is measuring the cytokine from the serum or by intracellular cytokine staining a better indicator of DC functions or activation? Analysis of the activation of vaccinated DCs showed a relationship with clinical benefit, and associated predictive markers of the most effective DCs have been reported ^115^. Given the limited consensus, ground-breaking developments in manufacturing, quality control, and optimized delivery of the vaccine to overcome the hurdle of cross-priming and TME are still needed.

A whole allogeneic pancreatic cell line secreting GM-CSF, called GVAX, is another strategy to use DCs *in vivo* that stimulate the accrual and function of APCs to the vaccine site. This strategy can potentially involve all DC subsets, not only the subsets in the apheresis product, and result in increased T-cell infiltration and development of tertiary lymphoid structures even in a so-called “non-immunogenic” tumor such as pancreatic cancer or prostate cancer ^143, 144^. This method has the merit of not involving the labor-intensive and expensive *ex vivo* manufacturing process. However, a phase IIb study of a combination of GVAX and a mesothelin-expressing live-attenuated listeria vaccine CRS-207 did not show improved overall survival ^145, 146^.

## Cancer vaccines in prophylaxis of cancer

Current data support the concept that cancer vaccines can induce more efficient immune responses and improved control of disease when the tumor burden is not overwhelmingly high, because targeting low tumor burden avoids immune suppressive effects of tumor cells and the cumulative impacts of cytotoxic therapies on immune function. In this sense, investigation of cancer vaccines in the prophylactic or preventive setting is reasonable, in both primary and secondary prevention ^147– 151^. However, because a prophylactic vaccine is used for healthy people rather than cancer patients who have exhausted other therapies, it is critical to avoid inducing autoimmune responses or other adverse effects. Thus, choice of antigens unique to tumors that are not expressed in normal tissue is essential. Intralesional delivery of a DC vaccine into ductal carcinoma *in situ* targeting HER2/neu showed a decline or disappearance of HER2/neu expression ^152^. Prophylactic vaccines in high-risk patients with Lynch syndrome or colorectal cancer with microsatellite instability (MSI) are under study. NCT01885702 is studying autologous DC vaccines loaded with CEA and specific frameshift-derived neoantigen peptides in patients with either colorectal cancer that is MSI-unstable or carriers of a germline MMR-gene mutation with no signs of cancer, who are HLA-A2.1 ^+^. Analysis of the blood from enrolled patients showed antigen-specific T cells against several neoepitopes, including epitopes from germline mutations, in immune function–related genes such as TGFβ receptor and caspases, supporting the rationale of a prophylactic role of cancer vaccines in high-risk patients, although final results are not yet posted. In addition, a considerable body of evidence suggests the sharing of some tumor antigens, such as hypoglycosylated MUC1, with other inflammatory diseases ^150, 153^. For example, epidemiologic studies suggest a reduced risk of breast cancer in patients with a history of mastitis. Clinical studies to test this hypothesis are under way.

## Vaccine delivery–related issues: routes of administration, accessibility of cancer vaccines, and schedule

Several routes of injection have been studied. Unlike vaccination against infectious organisms, the mucosal route has rarely been used. Most vaccines have been studied using a parenteral route, primarily subcutaneous, intradermal, or intramuscular, with less favor for intravenous delivery because of low immune response and concerns about direct organ toxicity or anaphylaxis. Intratumoral injection has been studied predominantly in melanoma and brain tumors often with local conditioning with cytokine or toxoid but also in several solid tumors in case of vectors of lytic or direct cytopathic potential ^29, 154, 155^. The immunologic properties and expected mechanism of immune activation have led investigators to individual choice among subcutaneous, intradermal, intramuscular injection, and intravenous infusion. In theory, intravenous infusion of DCs can deliver DCs to secondary lymphoid tissue rapidly and intratumoral injection has an advantage of modulating the TME. However, intradermal delivery of DCs was most effective in animal models ^156, 157^. In general, relatively few DCs were detected in the DLNs when they were intradermally or intratumorally administered. Direct injection into a single or multiple lymph nodes did not show any improvement compared with intradermal injection. Other systemic or local intervention, including cytokine or TLR agonist administration to improve the DC and T-cell homing or enhance local inflammation, might be helpful ^158^. Overall, the chemical and immunologic nature of the vaccine composition that can result in local tissue damage or altered immunologic cascade should be considered to determine the optimal route and the depth of injection to maximize safety and immunogenicity. Vaccine route may also affect the balance between responses of circulating versus tissue-resident memory T cells, as both may be necessary for efficacy ^124, 159, 160^.

Other than the vaccine platform–specific route of administration, there are issues related to vaccine delivery. Availability of cancer vaccines is one of those. Some vaccines are facility-dependent in their manufacturing or delivery, and it is hard to expand their use to the community setting outside cutting-edge research facilities. To expand the use of cancer vaccines successfully, the vaccine platform should be designed with the widening of accessibility in mind. Optimum interval spacing or doses of cancer vaccines are largely unknown because of a lack of information from clinically successful cancer vaccines in contrast to vaccines against infectious organisms.

## Summary

During the past 30 years of modern cancer vaccine development, various vaccine platforms have been tried in clinical trials based on successful preclinical models. Sources of tumor antigen can be synthetic (nucleic acids or peptides), viral, or microbial vectors or autologous or allogeneic tumor cell–derived cells, tumor cell lysates or RNA. Some platforms use infectious organisms to present the tumor antigen to APCs *in vivo*, whereas some researchers use DCs *in vitro* for antigen loading. Co-stimulatory molecules and cytokines have been tried, but no clear consensus has been made on the optimal choice. However, a prioritized set of immunomodulatory molecules to use as vaccine adjuvants has been developed by a National Cancer Institute working group ^161^. The majority of the vaccines that were successful in inducing immunologic anti-tumor response could not match that success in the clinical response, indicating the need for improved vaccine platforms and probably combinations with checkpoint inhibitors or other methods to block immune suppression by cancer.

## Conclusions

Cancer immunotherapy has established its position as the fourth pillar of anti-cancer therapy, complementing surgery, chemotherapy, and radiation. Following monoclonal antibodies against tumor antigens, checkpoint inhibitors, and adoptive cell therapy, cancer vaccine research is poised to achieve more breakthrough discoveries. Synergistic effects in combination therapy with other strategies will be critical when current data clearly indicate that the vaccine alone is not sufficient to control clinically advanced cancers. Not all tumors are immunogenic by themselves (“cold tumors”), and the use of cancer vaccines to turn the tumor “hot” by inducing immune responses that then can be amplified by blockers of negative regulatory immunologic signals will make “cold” tumors more susceptible to checkpoint blockade and other inhibitors of immunoregulatory mechanisms. Cancer vaccines can contribute to overcoming the current issues of lower-than-desired response rates and efficacy in current cancer immunotherapy regimens.
